# Thiamine as a putative natural modulator of PPARγ: exploring a nutrient-based approach for type 2 diabetes

**DOI:** 10.3389/fphar.2025.1712511

**Published:** 2025-11-24

**Authors:** Kalpana Panati, Parasuraman Aiya Subramani, Venkata Ramireddy Narala

**Affiliations:** 1 Department of Biotechnology, Government College for Men, Kadapa, India; 2 Fish diseases unit, Thünen Institute of Fisheries Ecology, Bremerhaven, Germany; 3 Department of Zoology, Yogi Vemana University, Kadapa, India

**Keywords:** type 2 diabetes, PPARγ, insulin sensitivity, inflammation, thiazolidinediones, thiamine analogues

## Abstract

The therapeutic targeting of peroxisome proliferator-activated receptor gamma (PPARγ) for type 2 diabetes (T2D) remains a double-edged sword: while thiazolidinediones are efficacious, their severe side effects necessitate the discovery of safer modulators. We propose a novel nutrient-centred hypothesis that thiamine (vitamin B1), an essential micronutrient, may act as a natural ligand for PPARγ. To investigate this, we adopted a translational approach. Molecular docking and dynamics simulations established that thiamine forms a stable, high-affinity interaction with the PPARγ ligand-binding domain. Functionally, in 3T3-L1 adipocytes, thiamine induced adipogenesis and PPARγ-response element binding with a potency analogous to rosiglitazone, suggesting direct agonistic activity. Corroborating these mechanistic insights at the clinical level, a new meta-analysis of randomized controlled trials demonstrates that high-dose benfotiamine, a synthetic thiamine derivative, significantly improves neuropathic and vascular outcomes in T2D patients. While the contribution of thiamine’s established antioxidant effects to these clinical benefits cannot be ruled out, the synergy of computational, cellular, and human evidence provides a compelling foundation for our hypothesis. This study suggests that thiamine could act as a PPARγ ligand and serve as a safer treatment option for metabolic disorders, which needs to be tested *in vivo*.

## Introduction

Diabetes mellitus is a chronic metabolic disease marked by sustained hyperglycaemia due to impaired insulin secretion, insulin resistance, or both, and is increasingly recognized as a state of low-grade chronic inflammation (O'Neill and Hardie, 2013; Ojo et al., 2023; Wellen and Hotamisligil, 2005). Type 2 diabetes (T2D), which accounts for over 90% of global cases, is rising at an alarming rate, particularly in low- and middle-income countries. Its pathogenesis involves a complex interplay of genetic, lifestyle, dietary, and environmental factors, leading to insulin resistance and progressive β-cell dysfunction (Laakso, 2019). Despite the availability of pharmacological agents, achieving durable glycaemic control and preventing complications remains a major challenge (Inzucchi et al., 2014). Current treatment strategies primarily target blood glucose regulation and insulin sensitivity, yet they often fall short in addressing the underlying pathophysiology or preventing long-term complications (Wilson et al., 2025; Zheng et al., 2018).

Peroxisome proliferator-activated receptor gamma (PPARγ), a nuclear hormone receptor, plays a key role in adipogenesis, lipid metabolism, glucose homeostasis, and inflammation (Kounatidis et al., 2025). PPARγ is a well-validated therapeutic target for improving insulin sensitivity and reducing inflammation, particularly in adipose and hepatic tissues (Camp, 2003). Full agonists, such as the thiazolidinediones (TZDs), are potent insulin sensitizers but their clinical utility is limited by significant side effects, driving the search for safer modulators, including natural ligands or selective PPARγ modulators (SPPARMs) that can dissociate beneficial metabolic effects from adverse outcomes (Nesto et al., 2004). Consequently, there is growing interest in identifying safer, better-tolerated compounds—particularly natural or endogenous ligands—that can modulate PPARγ activity (Wang et al., 2014; Derangula et al., 2023). Whether thiamine can truly function as a SPPARM remains to be determined, since its observed effects also involve pathways regulated by PPARα.

Thiamine (vitamin B1) is an essential micronutrient, well-known for its role as a cofactor in key enzymes of carbohydrate metabolism (Bozic and Lavrnja, 2023). Importantly, thiamine deficiency is prevalent in T2D and has been linked to its complications, particularly neuropathy, likely through mechanisms involving oxidative stress and impaired endothelial function (Page et al., 2011; Ziegler et al., 2023). While high-dose thiamine supplementation has shown promise in improving these complications, its effects on core metabolic parameters, such as insulin sensitivity, have been inconsistent, suggesting that its benefits may be mediated through pathways independent of classic insulin-sensitizing mechanisms.

Given thiamine’s established clinical benefits in diabetic complications, yet unclear molecular mechanism of action, we hypothesized that it might influence signalling pathways relevant to metabolic homeostasis. To strengthen this rationale, we conducted a focused meta-analysis of randomized controlled trials evaluating benfotiamine supplementation in T2D. This analysis, performed as part of the present study, demonstrated consistent clinical improvements in neuropathic and vascular outcomes, aligning with biological processes regulated by PPARγ. These findings provided translational support for exploring thiamine as a potential natural activator of PPARγ and a nutrient-based therapeutic candidate for metabolic disorders.

Recent clinical trials with benfotiamine, a lipid-soluble thiamine analogue, have provided consistent evidence for its efficacy in alleviating diabetes-related complications such as neuropathy and vascular dysfunction. These randomized, placebo-controlled trials demonstrated that benfotiamine supplementation—at doses ranging from 400 to 1,050 mg/day—significantly improved neuropathic symptoms, endothelial function, and markers of oxidative stress (Mallika et al., 2025; Stirban et al., 2006; Haupt et al., 2005; Alkhalaf et al., 2010; Fraser et al., 2012; Alkhalaf et al., 2012; Stirban et al., 2013; Bönhof et al., 2022; Stracke et al., 2008). These clinical benefits, although traditionally attributed to enhanced transketolase activity and reduced glycation, align closely with mechanisms known to follow PPARγ activation, such as improved insulin sensitivity and anti-inflammatory signalling.

Collectively, these findings suggest that thiamine and its derivatives may exert broader transcriptional control over metabolic and inflammatory pathways, warranting mechanistic investigation into their potential role as natural PPARγ modulators.

Therefore, this study aims to investigate systematically, for the first time, the hypothesis that thiamine may act as a direct modulator of PPARγ activity, potentially representing a novel, nutrient-based approach to influencing metabolic signalling.

## Preliminary evidence

The rising prevalence of T2D poses a significant global health challenge, particularly among populations that rely on polished white rice as a primary dietary staple. The cultural practice of rice polishing, which removes the nutrient-rich bran and germ, significantly reduces thiamine content, along with fibre and other bioactive compounds. This process increases the glycaemic index of rice, contributing to elevated blood glucose levels and insulin resistance. Epidemiological evidence suggests a correlation between high consumption of polished rice and an increased incidence of T2D in specific populations, highlighting the need for nutrient-centred strategies to address this growing metabolic disorder (Bhavadharini et al., 2020; Mohan et al., 2009).

Several studies have explored the therapeutic potential of thiamine and its derivatives, such as benfotiamine, in managing T2D, with a focus on their ability to address metabolic dysfunctions, oxidative stress, and vascular complications. Benfotiamine, a more bioavailable analogue of thiamine, has been widely investigated in clinical settings due to its enhanced absorption and efficacy in counteracting diabetic complications like neuropathy, nephropathy, and endothelial dysfunction. For example, a double-blind, randomized, placebo-controlled trial involving patients with diabetic nephropathy showed that benfotiamine significantly improved thiamine status, though it did not markedly alter kidney function markers over the study period. Another clinical trial demonstrated that chronic benfotiamine treatment restored endothelial function in T2D patients, highlighting its role in preventing postprandial vascular impairments. Systematic reviews have further supported the benefits of thiamine supplementation in improving glycaemic outcomes and reversing diabetes-related cardiovascular dysfunctions, with doses ranging from 100 to 900 mg/day showing promise in reducing fasting blood glucose and oxidative stress. These findings underscore the growing interest in thiamine-based interventions as adjunct therapies for T2D. Intriguingly, recent research has established thiamine itself as a natural PPARγ ligand through *in silico* docking, molecular dynamics simulations, and *in vitro* adipogenesis assays, providing a mechanistic basis for its antidiabetic effects (Subramani et al., 2022).

Although direct experimental evidence for PPARγ activation by thiamine is currently limited, several studies provide indirect mechanistic support. Thiamine and its derivatives have been reported to suppress inflammation by inhibiting NF-κB and modulating redox-sensitive signalling pathways (Bozic and Lavrnja, 2023; Balakumar et al., 2010; Das et al., 2021), both of which act upstream of PPARγ. Such indirect regulatory effects reinforce our central hypothesis that thiamine may function as a natural activator of PPARγ. These observations collectively strengthen the theoretical foundation for exploring thiamine-mediated PPARγ modulation in T2D.

## Thiamine and PPARα signalling

While the present study focuses on thiamine as a potential activator of PPARγ, we also recognize the relevance of PPARα in metabolic regulation. PPARα primarily governs lipid oxidation and energy balance, and several synthetic dual PPARα/γ agonists have shown therapeutic promise in improving both glucose and lipid metabolism with fewer side effects than selective PPARγ agonists (Chinetti-Gbaguidi et al., 2005; Staels and Fruchart, 2005).

Thiamine and its derivative benfotiamine have been reported to enhance fatty acid β-oxidation and improve lipid homeostasis (Alkhalaf et al., 2010; Alkhalaf et al., 2012), suggesting that partial activation or crosstalk with PPARα cannot be excluded. This observation aligns with the concept of nutrient-based dual PPARα/γ modulation, offering an additional mechanistic dimension to thiamine’s metabolic benefits. Further studies are warranted to clarify whether thiamine’s regulatory effects extend to PPARα-dependent pathways.

## Hypothesis

We hypothesize that thiamine exerts its antidiabetic effects primarily through direct activation of PPARγ, functioning as a potential natural ligand (Subramani et al., 2022). Through this activation, thiamine may enhance insulin sensitivity, improve glucose utilization, and suppress systemic inflammation. In parallel, thiamine may also influence upstream regulatory pathways—such as AMP-activated protein kinase (AMPK), Sirtuin 1, and NF-κB—which converge on PPARγ activation and reinforce its downstream metabolic and anti-inflammatory effects (Feng et al., 2019).

Consistent with this hypothesis, emerging *in vitro* and *in vivo* evidence suggests that thiamine and its derivatives (e.g., benfotiamine) modulate gene expression networks involved in glucose uptake, adipocyte differentiation, and cytokine regulation (Cassiano et al., 2022). Activation of PPARγ by thiamine may upregulate canonical target genes, such as GLUT4, adiponectin, and adipocyte fatty acid-binding protein (aP2), while suppressing proinflammatory mediators including TNF-α and IL-6.

**FIGURE 1:**
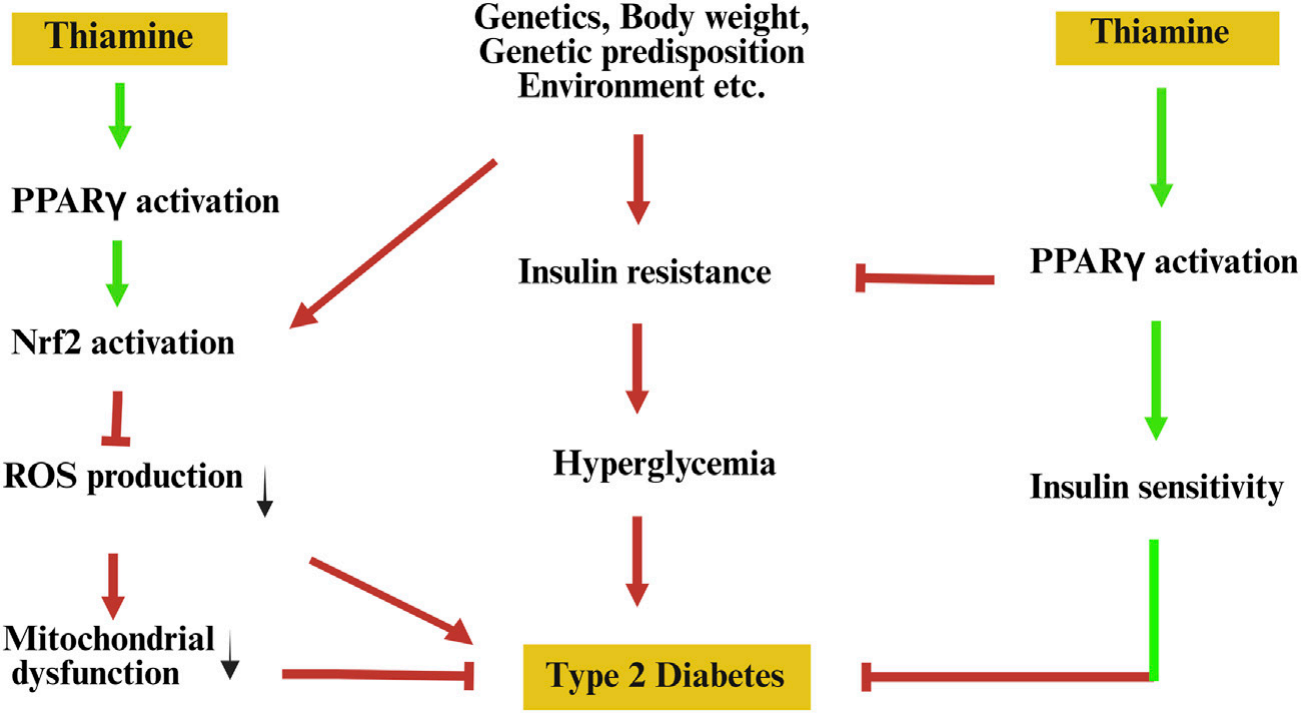
Hypothetical model illustrating the potential role of thiamine in reprogramming PPARγ signalling to prevent type 2 diabetes. Pathological factors such as genetic predisposition, environmental influences, and obesity contribute to insulin resistance, mitochondrial dysfunction, and elevated oxidative stress, all promoting the onset of type 2 diabetes. Thiamine is proposed to activate peroxisome proliferator-activated receptor gamma (PPARγ), leading to downstream activation of nuclear factor erythroid 2-related factor 2 (Nrf2), which reduces reactive oxygen species (ROS) production and alleviates mitochondrial dysfunction. Concurrently, PPARγ activation enhances insulin sensitivity, thereby mitigating insulin resistance and hyperglycaemia—key contributors to the pathogenesis of type 2 diabetes. Green arrows indicate proposed beneficial effects; red arrows and T-bars represent detrimental pathways and inhibitory interactions.

This hypothesis diverges from current thinking by proposing that thiamine is not merely a metabolic cofactor or antioxidant, but also a functional modulator of transcriptional activity through direct or indirect influence on PPARγ signalling pathways (Figure 1). This perspective bridges two previously separate domains: micronutrient-based interventions and nuclear receptor pharmacology.

## Evolution of hypothesis

The hypothesis that thiamine modulates PPARγ activity stems from emerging insights into its multifaceted role beyond its classical function as a coenzyme in carbohydrate metabolism. Recent studies suggest that thiamine and its derivatives may exert transcriptional regulatory effects relevant to glucose homeostasis, inflammation, and adipocyte biology—domains traditionally governed by PPARγ signalling.

### Supporting evidence

Benfotiamine has been shown to inhibit the activation of proinflammatory transcription factors such as NF-κB and to reduce oxidative stress in diabetic models (Balakumar et al., 2010). These pathways are tightly linked to insulin resistance and are recognized as upstream regulators of PPARγ (Das et al., 2021). *In vitro* studies using adipocyte and macrophage models have demonstrated that thiamine can influence gene expression profiles associated with glucose uptake and inflammatory response—effects typically observed upon PPARγ activation (Bozic and Lavrnja, 2023; Hammes et al., 2003). Furthermore, thiamine treatment has been reported to increase adiponectin levels and GLUT4 expression in rodent models, which are canonical downstream targets of PPARγ signalling (Tanaka et al., 2010; Rad et al., 2024).

While direct ligand-binding assays demonstrating thiamine’s affinity for PPARγ are lacking, some computational docking studies suggest potential for thiamine or its phosphorylated metabolites to interact with nuclear receptor ligand-binding domains (Subramani et al., 2022). Additionally, AMPK and SIRT1—both implicated in thiamine’s mechanism of action—have been shown to enhance PPARγ coactivator activity indirectly (Cantó and Auwerx, 2009; Kalyesubula et al., 2021), suggesting a plausible mechanistic bridge.

### Inconclusive evidence

Despite these associative findings, no direct biochemical evidence currently confirms that thiamine or its metabolites serve as *bona fide* PPARγ agonists. Most of the beneficial effects attributed to thiamine in diabetes models have been explained by its antioxidant and endothelial-stabilizing properties rather than its transcriptional modulation. Moreover, clinical trials investigating thiamine or benfotiamine in diabetic patients have yielded inconsistent results regarding glycaemic control, although they frequently report improvements in neuropathy and endothelial function (Stracke et al., 2008; Ang et al., 2008). These observations suggest that thiamine’s primary therapeutic value may lie in mitigating diabetes complications rather than core glucose regulatory mechanisms—a challenge to the proposed hypothesis. Furthermore, studies that measure PPARγ activity (e.g., reporter assays or coactivator recruitment) in the presence of thiamine are scarce, limiting conclusive interpretation.

### Integrative perspective

Given the current state of evidence, it is plausible that thiamine influences PPARγ signalling via indirect mechanisms—such as redox-sensitive transcriptional control, AMPK-SIRT1 crosstalk, or suppression of inflammatory pathways—rather than acting as a direct ligand. This perspective aligns with an emerging view that metabolic cofactors and micronutrients may serve as modulators of nuclear receptor function through upstream regulatory networks rather than through classical receptor-ligand interactions (Patti, 2004). As such, thiamine may act as a functional enhancer of PPARγ signalling under pathological conditions such as oxidative stress or chronic inflammation, which are hallmarks of T2D.

Thus, we propose rigorous experimental testing to determine whether thiamine’s effects on PPARγ are direct, indirect, or co-regulated via redox or energy-sensing pathways such as AMPK or SIRT1.

Further experimental validation—particularly studies employing PPARγ reporter assays, knockdown or antagonist models, and transcriptomic profiling in the presence of thiamine—is warranted to clarify this relationship. The hypothesis remains compelling, as it offers a novel conceptual framework linking micronutrient biology to nuclear receptor pharmacology, but it must be rigorously tested against the mechanistic and structural constraints of receptor biology.

## Testing of the hypothesis

To robustly evaluate the hypothesis that thiamine functions as a natural PPARγ agonist and ameliorates T2D, we propose a three-tiered, integrated experimental strategy encompassing molecular, cellular, genetic, and *in vivo* investigations. This approach aims to delineate the mechanistic basis, therapeutic potential, and genetic relevance of thiamine action in the context of T2D.

### Molecular and cellular validation of thiamine as a PPARγ agonist

To directly assess whether thiamine activates PPARγ, a combination of biophysical binding assays, reporter gene assays, and gene expression profiling can be employed. *Ligand-binding assays:* A fluorescence polarization or surface plasmon resonance assay can be performed using purified human PPARγ ligand-binding domain to evaluate the binding kinetics and affinity of thiamine relative to rosiglitazone (Nolte et al., 1998). This is a more sensitive and high-throughput approach than classical radiolabelled binding. *Transcriptional activation assays:* HEK293T or COS-7 cells can be transfected with a PPARγ-responsive luciferase reporter plasmid (e.g., PPRE-Luc) and with PPARγ expression vectors. Thiamine treatment can be compared with rosiglitazone and evaluated in the presence or absence of the PPARγ antagonist GW9662 (Dreier et al., 2017). *Gene expression profiling:* In 3T3-L1 preadipocytes, thiamine-induced differentiation can be confirmed via Oil Red O staining, and mRNA levels of canonical PPARγ target genes (e.g., *aP2*, *adiponectin*, *lipoprotein lipase*) can be quantified by qPCR and validated by RNA-seq for global transcriptomic analysis (Tontonoz et al., 1995). *Functional dependence studies:* CRISPR/Cas9-mediated knockouts of SLC19A2 (thiamine transporter) and PPARγ in 3T3-L1 or human adipose-derived stem cells can test if thiamine-induced effects on adipogenesis and gene expression are transporter- and receptor-dependent. Rescue experiments with thiamine pyrophosphate (TPP) or overexpression constructs can validate specificity. These studies can establish whether thiamine acts as a *bona fide* PPARγ agonist, distinct from its classical role in metabolism.

### *In vivo* evaluation in preclinical models of T2D

The WNIN/GR-Ob rat model of polygenic obesity-induced T2D can be utilized to assess the therapeutic potential of thiamine *in vivo* (Harishankar et al., 2011). Animals may receive oral thiamine (5–100 mg/kg) ± GW9662 for 8–12 weeks, with rosiglitazone-treated groups as controls. Glycaemic indices (e.g., glucose, HbA1c, OGTT, ITT, HOMA-IR), glycogen content, and insulin signalling proteins can be assessed. Adipose histology and markers of mitochondrial biogenesis (PGC-1α, TFAM) and fat browning (UCP1) can be analyzed. Oxidative stress markers in key tissues can also be measured. PPARγ dependency of thiamine effects can be evaluated using 2-way ANOVA. Alternative models, such as STZ-induced diabetic mice or db/db mice, may be used to validate reproducibility across T2D phenotypes.

### Genetic and functional correlation in human populations

To extend findings to a clinical context, a population-based case-control study can explore the genetic determinants of thiamine responsiveness in T2D patients. A cohort of T2D patients and age-/sex-matched healthy controls can be genotyped for SNPs in *SLC19A2* (encoding thiamine transporter 1, which is essential for transporting thiamine into cells), particularly in exon 2 and the promoter region, using Sanger sequencing or high-throughput targeted NGS. Exploratory analyses may investigate associations between *SLC19A2* polymorphisms and PPARγ expression or methylation, offering insights into gene–nutrient–receptor interactions.

This multi-tiered experimental strategy employing state-of-the-art biochemical, genetic, and *in vivo* approaches can be designed to rigorously test the novel hypothesis that thiamine is a functional PPARγ activator with therapeutic relevance to T2D. Such work has the potential to redefine thiamine as a metabolic modulator beyond its classical vitamin role.

### Differentiation of metabolic vs. PPARγ-mediated activity

While our *in silico* docking, molecular dynamics and *in vitro* adipogenesis data are consistent with direct interaction of thiamine with the PPARγ ligand-binding domain, these findings do not exclude indirect activation mediated by thiamine’s canonical metabolic roles (for example, conversion to TPP and consequent alterations in cellular energy/redox state). To address this, future work can include direct biophysical binding assays (surface plasmon resonance and/or isothermal titration calorimetry and fluorescence polarization) using purified PPARγ-LBD to obtain binding constants, together with PPARγ PPRE-luciferase reporter assays in cultured cells treated with or without the PPARγ antagonist GW9662 to test whether transcriptional activation is PPARγ dependent. To dissociate metabolic from ligand-like effects, this can be achieved by comparing responses to thiamine and non-phosphorylated thiamine analogues, and testing whether pharmacologic or genetic blockade of thiamine phosphorylation attenuates reporter activation.

### Bioavailability and transporter dependence assays

Thiamine and its phosphorylated derivatives are hydrophilic and rely on specific transporters (SLC19A2 and SLC19A3) for cellular uptake (Gabriel et al., 2024). The lipid-soluble prodrug benfotiamine more readily crosses membranes and is commonly used in clinical studies to raise intracellular thiamine pools. Therefore, it is expected that transporter expression and prodrug formulation can strongly influence intracellular availability and any nuclear receptor engagement. Experiments in cell lines with SLC19A2 knockdown or overexpression to determine whether PPARγ activation by thiamine is transporter-dependent, and to measure intracellular thiamine and TPP concentrations by HPLC as correlates of activity.

## Implications

If proven, the potential of thiamine to activate PPARγ could revolutionize diabetes treatment by offering a safer alternative to current PPARγ agonists, with fewer side effects. This could open up new therapeutic pathways for managing T2D and addressing complications such as oxidative stress and inflammation.

### Therapeutic implications

One of the most compelling therapeutic implications of this hypothesis is the potential of thiamine as an adjunct or even a substitute for TZDs, a class of drugs that act as PPARγ agonists. This would not only expand the therapeutic options available but also potentially minimize the long-term cardiovascular risks associated with TZD use. Furthermore, thiamine-based PPARγ modulators can be developed, focusing on tailored pharmacodynamics to improve tissue selectivity and minimize systemic side effects. Such compounds could have a broader therapeutic profile, modulate glucose and lipid metabolism, and preserve normal tissue homeostasis.

### Impact on diabetic complications

The potential role of thiamine in mitigating diabetic complications is particularly noteworthy. Diabetes is associated with several long-term complications, including neuropathy, nephropathy, and cardiovascular diseases (Rabbani and Thornalley, 2011). These complications are partly driven by oxidative stress and inflammation, both of which can be modulated by thiamine. As an essential cofactor for enzymes involved in antioxidant defence (e.g., transketolase), thiamine can reduce reactive oxygen species production, thereby protecting tissues vulnerable to diabetic complications against oxidative damage (Beltramo et al., 2021). Moreover, thiamine’s effects on PPARγ may also exert anti-inflammatory actions, another critical factor in preventing diabetic complications. It is plausible that thiamine could synergize with other established treatments for T2D, such as metformin and resveratrol, to improve outcomes. Metformin, for example, primarily acts by activating AMPK, which complements the activation of PPARγ in regulating glucose and lipid metabolism. The dual effect of thiamine, in combination with other nutrients or drugs, may enhance therapeutic efficacy, further supporting its potential as an adjunctive therapy in diabetes management.

### Broader applications

The therapeutic potential of thiamine extends beyond T2D and could benefit a broader range of metabolic disorders. For example, thiamine’s ability to modulate lipid and glucose metabolism makes it a promising candidate for the treatment of metabolic syndrome, a condition characterized by insulin resistance, dyslipidaemia, and abdominal obesity. In animal models, thiamine supplementation has shown potential to improve insulin sensitivity and reduce visceral fat accumulation, both of which are key features of metabolic syndrome (Serra et al., 2025). Similarly, thiamine could play a role in the management of non-alcoholic fatty liver disease (NAFLD), a common comorbidity of T2D. Given the close link between NAFLD, insulin resistance, and abnormal lipid metabolism, thiamine’s effects on PPARγ and its antioxidant properties may help reduce hepatic steatosis and inflammation (Nogueira and Cusi, 2024). These benefits suggest a potential therapeutic avenue for managing NAFLD and preventing its progression to cirrhosis or hepatocellular carcinoma (Pan et al., 2022).

Additionally, the potential impact of thiamine on obesity is noteworthy. As a cofactor in the pentose phosphate pathway, thiamine plays a key role in cellular energy metabolism and the regulation of adipogenesis. Thiamine’s modulation of PPARγ could influence adipocyte differentiation and lipid storage, which may prove beneficial in addressing the increasing global burden of obesity. Thus, thiamine-based therapies may have a wide-ranging effect on multiple aspects of metabolic health.

### Limitations and counterarguments

Despite its potential, the hypothesis linking thiamine to PPARγ activation and T2D management has several limitations and counterarguments that must be addressed. First, while *in vitro* and animal data suggest that thiamine can activate PPARγ, there is currently no direct evidence in human tissues demonstrating this effect. Clinical trials are necessary to validate the molecular findings and to determine whether the observed effects in animal models translate to human populations.

Another limitation is the potential risk of over supplementation. While thiamine is water-soluble and generally considered safe, high-dose supplementation over extended periods may pose risks, particularly in individuals with impaired renal function or those taking other medications. The long-term safety profile of high-dose thiamine remains unclear; therefore, careful monitoring of treatment duration and dose optimization is essential. Personalized treatment strategies based on genetic factors, such as variations in the *SLC19A2* gene, may help minimize risks and improve therapeutic outcomes.

## Conclusion

In conclusion, the hypothesis that thiamine can act as a natural PPARγ activator and a potential therapeutic agent for T2D and related metabolic disorders holds significant promise. The therapeutic implications, including its potential as a substitute for TZDs and its role in preventing diabetic complications, position thiamine as a valuable adjunct or alternative in the management of diabetes. Thiamine responsiveness may vary based on genetic polymorphisms, suggesting the potential for future precision nutrition approaches. However, further molecular validation, clinical trials, and careful consideration of safety and dosage are necessary to realise its therapeutic potential fully. Short-term biomarker studies, mediator analyses, and randomized mechanistic sub-studies are recommended for validating the proposed mechanisms and strengthening translational relevance. The proposed paradigm shift toward nutrient-centred approaches could lead to more sustainable and effective strategies for managing chronic diseases, offering hope for a future where nutritional interventions play a significant role in preventing and treating diabetes and its complications.

## Data Availability

The original contributions presented in the study are included in the article/supplementary material, further inquiries can be directed to the corresponding author.
